# Supplementary figures and images for: The effect of different anesthetics on the incidence of AKI and AKD after neurosurgical procedures
Source: PLoS One. 2024 Dec 31;19(12):e0315295. doi: 10.1371/journal.pone.0315295 (PMC11687668; doi:10.1371/journal.pone.0315295)

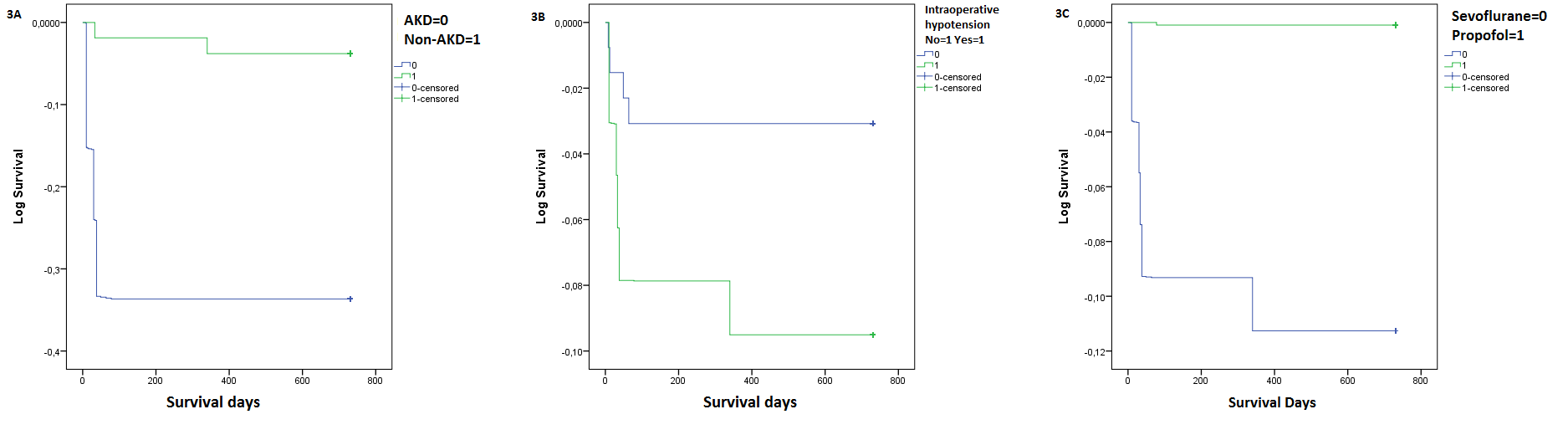

Supplement: S1 Fig — A-C. Kaplan-Meier analysis of survival probability according to AKD versus non-AKD (A), presence of intra-operative hypotension (B) and according to propofol versus sevoflurane (C). (TIF) [file pone.0315295.s001.tif]
