# Supplementary material for: The effect of different anesthetics on the incidence of AKI and AKD after neurosurgical procedures
Source: PLoS One. 2024 Dec 31;19(12):e0315295. doi: 10.1371/journal.pone.0315295 (PMC11687668; doi:10.1371/journal.pone.0315295)
Supplement: Attachment — Submitted filename: Response to Reviewers PLOS ONE(1).docx [file pone.0315295.s003.docx]

Zagreb, Croatia, October 29 2024

Dear Editor,

Please see the revised version of our manuscript (PONE-D-24-18913R1) entitled "The effect of different anesthetics on the incidence of AKI and AKD after neurosurgical procedure." We believe the revisions based on this peer-review exercise have significantly improved the quality of our manuscript.

All authors have read and approved the submission of the manuscript; the manuscript has not been published and is not being considered for publication elsewhere, in whole or in part, in any language.

We hope you will find our study and obtained results interesting to be considered for publication in your prestige journal especially after changes which hopefully made the manuscript easier to comprehend.

**Reviewer #1: The Authors have worked hard to improve this article. Although of potential interest to Plos One readership, the manuscript still requires minor modifications before it can be considered for publication.**

**Abstract. AKI acronyms should be written. And further on in the abstract, acute kidney injury should be changed in AKI**

As suggested, we have changed acute kidney injury to AKI in the whole abstract.

**Abstract. Please include the incidence of AKI and AKD in your cohort.**

As suggested, we have included the incidence of AKI and AKD in the abstract.

**The following sentence should be rewritten:**

**"The incidence of moderate to severe AKI was low in our cohort despite including patients with CKD, who are more prone to develop AKI episodes"**

**correct sentence:**

**"The incidence of moderate to severe AKI was low in our cohort probably due to the exclusion of patients with CKD, who are more prone to develop AKI episodes"**

We have rewrite the sentence as suggested by the reviewer.

**I would include a sentence in your Limitations: Approximately one-quarter of the initial cohort of patients undergoing neurosurgical procedures did not have an available postoperative creatinine value. Although this data does not differ significantly from the literature [Villa G et al, 2021], this significant proportion of excluded patients and the exclusion of patients with CKD does not allow us to have real data on the incidence of AKI and AKD in this setting.**

**Villa G, De Rosa S, Scirè Calabrisotto C, Nerini A, Saitta T, Degl'Innocenti D, Paparella L, Bocciero V, Allinovi M, De Gaudio AR, Ostermann M, Romagnoli S. Perioperative use of serum creatinine and postoperative acute kidney injury: a single-centre, observational retrospective study to explore physicians' perception and practice. Perioper Med (Lond). 2021 May 25;10(1):13.**

We have included the proposed sentence in the limitations section of the manuscript and added the suggested reference.

**Reviewer #2: Dear Author,**

**I have reviewed the revised version of your paper. While I appreciate your efforts to respond to reviewers' comments, I have come other additional observations:**

**1. Please define AKD in the introduction.**

We have moved the definition of AKD from methods to the introduction section of the manuscript as suggested by the reviewer.

**2. There appears to be an inconsistency with the definition of AKI used in this paper. It seems that you have applied the RIFLE classification (including for defining AKI severity), whereas, in response to Reviewer 3’s critique, the text was modified to indicate the application of KDIGO criteria. It would be more accurate to clarify the specific AKI criteria actually employed and to discuss the rationale and potential limitations of this choice in the Discussion section.**

We agree with the reviewer. We have put back the exact criteria we have used for the definition of AKI and added the rationale and potential limitations of this choice in the limitations/discussion section of the manuscript.

Discussion: Lines: 363-372

While some may view the reliance on RIFLE as a limitation, it is noteworthy that research comparing the performance of AKIN, KDIGO, and RIFLE criteria has shown that RIFLE can effectively identify a higher percentage of patients in the early stages of AKI [44].

Additionally, studies have indicated that the RIFLE criteria possess excellent discriminatory power for predicting mortality compared to the other two definitions. This suggests that, despite being an older classification system, RIFLE may still provide valuable insights in certain clinical contexts, particularly in identifying patients at risk for adverse outcomes early in the course of AKI.

Ultimately, the choice of criteria may depend on the specific objectives of a study or clinical assessment, and it is essential to consider the strengths and limitations of each classification system when interpreting results.

**3. It would be helpful to specify the type of statistical tests performed and to include the statistical values for the multivariate analyses added to the text (lines 260-262 and 271-273).**

We have specified the type of statistical tests and include statistical values for the multivariate analyses as suggested by the reviewer.

**Reviewer #3: I have no further comments, the authors have made the necessary revisions. I appreciate the effort and the response from the authors.**

Finally, we would like to thank all the reviewers and the editor for their critical appraisal, comments, and suggestions. Following several implemented changes, we hope that the current version of the manuscript can be considered for publication.

Sincerely

Vedran Premuzic
